# Computational-fitting method for mobility extraction in GaN HEMT

**DOI:** 10.1039/d3ra06630d

**Published:** 2023-11-07

**Authors:** Kuan-Chang Chang, Xibei Feng, Huangbai Liu, Kai Liu, Xinnan Lin, Lei Li

**Affiliations:** a School of Electronic and Computer Engineering, Peking University Shenzhen Graduate School Shenzhen 518055 China lilei@pkusz.edu.cn; b School of Integrated Circuits, Anhui Polytechnic University Anhui 241060 China xnlin@mail.ahpu.edu.cn

## Abstract

The third-generation semiconductor gallium nitride (GaN) has drawn wide attention due to its high electron mobility property. However, the classical mobility calculation methods such as Hall effect and transfer length method have limitations in accurately extracting the mobility of GaN High Electron Mobility Transistor (HEMT) due to their inability to consider the resistance in non-gate region or their high fabrication costs. This work proposes an effective yet accurate computational-fitting method for extracting the mobility of GaN HEMT. The method consists of measuring the total resistance between source and drain at different gate voltages over a very small range of overdrive voltage variations, when the sum of the transconductance and capacitance of the device is regarded as constants, and fitting a unique function of the total resistance with respect to the overdrive voltage to determine the carrier mobility and the non-gate resistance. The feasibility and reliability of the method has been also verified.

## Abstract 2

A computational fitting method, where *R*_SD_ is related to overdrive-voltage, is demonstrated for determining mobility in GaN HEMT.

## Graphical abstract

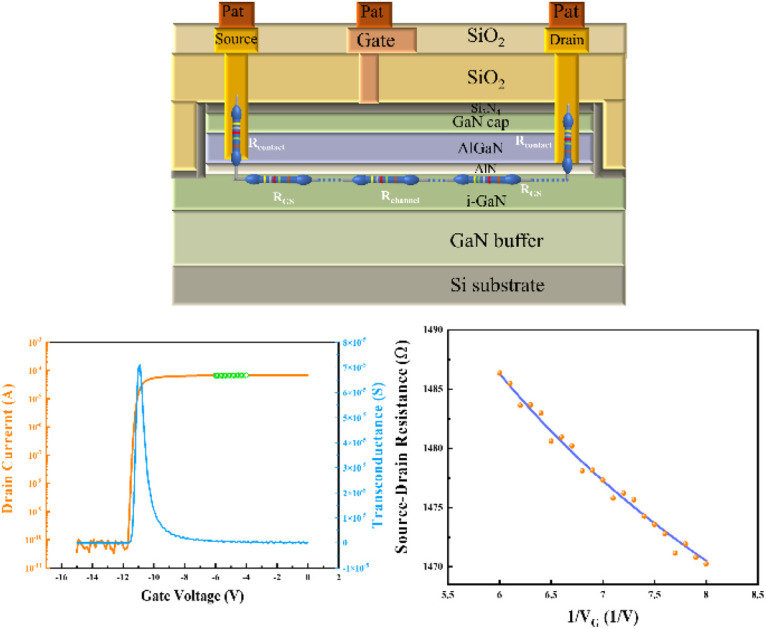

## Introduction

The extraction of electrical parameters is crucial for understanding the physical phenomena underlying the operation of transistors.^1–3^ Among these parameters, mobility and contact resistance play a critical role in assessing the performance of various transistors.^4–6^ The exact extraction of mobility and contact resistance can not only optimize the design and manufacturing process, improve the performance and reliability of transistors, but also provide important data for the study of the physical and electrical properties of materials, and help the research of materials science and electronic engineering.^7,8^ However, when dealing with different device structures, significant errors may arise when using the deformation of the classical Sachtang equation to extract these parameters, especially in the case of devices with considerable size and long distance between the gate and drain electrodes.^9^

The extraction of carrier mobility using the Shichman–Hodges intensive model in the linear region has been a widely used and straightforward method in silicon-based MOSFETs.^10,11^ Although this model does not account for the effect of non-gate region resistance, it can still be applied to the source-drain parasitic resistance of very small silicon-based MOSFETs.^12,13^ However, Gallium nitride high electron mobility transistor (GaN HEMT) has a large non-gate region, making it impossible to ignore the effect of non-gate-down resistance. Because of the wide bandgap, high electron mobility and other excellent physical material characteristics of GaN semiconductor, GaN HEMT has become a preferred choice for power amplifiers that require high mobility.^14–17^ Therefore, it is crucial to accurately calculate and extract mobility to evaluate the performance of GaN HEMT.^18,19^ However, the mobility calculated using the Shichman–Hodges model will be affected by the large non-gate resistance and will no longer be accurate. In this case, the most straightforward solution is to calculate the contact resistance or to exclude its effect to accurately extract the mobility.

The Hall effect method and transfer length method (TLM) are two of the most common methods for evaluating contact resistance and mobility, and are also applied to calculate the carrier mobility of GaN devices. However, each of these methods has limitations in application. The Hall effect method can only be applied to the materials that has not been made into finished products, meaning that it cannot be used to measure parameters of finished devices.^20–22^ The TLM method is the most widely used method, which requires specific test areas with varying pitches on the wafer, and the parameters are derived from linear fitting results.^23–26^ However, it is susceptible to process non-uniformity and has specific design and flow requirements. Thus, these classical methods have higher process requirements and more complex calculations, resulting in lower accuracy in extracting mobility, compared to the direct extraction of contact resistance and mobility from the completed device. Therefore, the development of alternative and more accurate techniques for extracting these parameters remains an important area of research in the field of transistor characterization.

In order to find a simple and cost-effective carrier mobility extraction method, this work proposes a computational-fitting method that is based on the fundamental equation and electrical measurements that can accurately extract the mobility of the device independent of the non-gate resistance. This method can exclude the influence of non-gate resistance and is particularly useful for addressing the challenges associated with GaN HEMTs where precise mobility analysis is difficult due to high non-gate resistance. Notably, the method proposed exhibits adaptability to a variety of materials, encompassing a wide range of emerging materials. Furthermore, the reliability and stability of this method are also verified through multiple experimental designs.

## Results and discussion

**Fig. 1:**
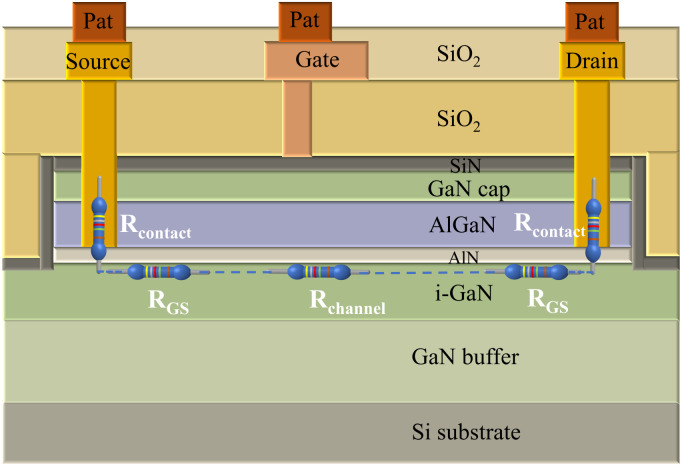
Schematic illustration of GaN-HEMTs device structure and the composition and distribution of total source–drain resistance.

Silicon-based GaN-HEMTs were chosen for demonstrating and validating the proposed mobility extraction method because of their large size, particularly the large gate-drain distance, which results in excessive non-gate resistance and makes it challenging to obtain accurate mobility using traditional methods. The GaN-HEMTs were fabricated as shown in Fig. 1. The GaN single crystal with (111) orientation was grown on the Si substrate to serve as buffer layer. Subsequently, the AlGaN/AlN/i-GaN heterostructure was synthesized using the metal–organic chemical vapor deposition (MOCVD) method. The AlGaN layer was 24.5 nm thick, and the AlN layer measured approximately 0.7 nm in thickness. The cap layer was GaN (3 nm) and the gate dielectric layer was Si_3_N_4_ (35 nm). SiO_2_ (300 nm) was used as passivation layer to isolate each device.

**Fig. 2:**
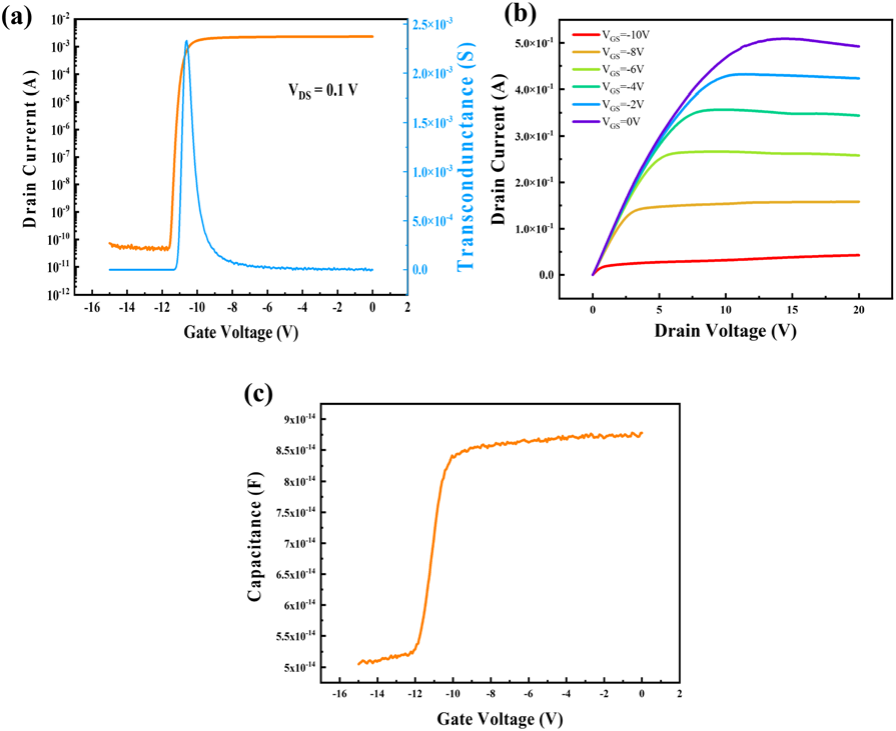
The basic electrical performance of GaN-HEMTs. (a) Transfer characteristic measured at 0.1 V drain voltage. (b) Output characteristics with gate voltage ranging from −10 V to 0 V. (c) *C*–*V* characteristics measured at the frequency of 100 kHz.

Fig. 2 displays the measured transfer and output characteristics, as well as stable capacitance–voltage (*C*–*V*) characteristics, which are crucial for the proposed extraction method.

The total source-drain resistance of GaN HEMT consists of the sub-gate resistance and the non-gate region resistance, which can be expressed as:

1 *R*_SD_ = 2*R*_contact_ + *R*_GS_ + *R*_GD_ + *R*_channel_

*R*_channel_ is the channel resistance under the gate, which is controlled by the gate voltage. *R*_contact_ is the contact resistance. *R*_GS_ is the resistance between gate and source. *R*_GD_ is the resistance between gate and drain. These resistances have constant magnitudes under DC stability and can therefore be treated as constants, which can be expressed as:

2 *R*_constant_ = 2*R*_contact_ + *R*_GS_ + *R*_GD_

Therefore, the influence of *R*_contact_, *R*_GS_, *R*_GD_ can be excluded by treating them as a constant *R*_constant_. As a result, the total resistance between source and drain can be expressed as

3 *R*_SD_ = *R*_constant_ + *R*_channel_

The basic current equation could be expressed as

4 Δ*I* = Δ*QμE*

where Δ*Q* is the number of carriers passing through a unit section, *E* is the intensity of electric field, and *μ* is the carrier mobility. The transfer characteristic was measured at *V*_D_ = 0.1 V, which is at least one order of magnitude lower than *V*_GS_ allowing the voltage on gate capacitor to be approximately equal to *V*_GS_. Based on this test setup, it is evident that the current variation is stable with the stability of transconductance, and the charge variation is stable with the stability of capacitance. Given that

5 Δ*Q* = *C*_ox_Δ*V*_GS_

6 Δ*I* = *g*_m_Δ*V*_Gs_

The current equation could be transformed into7
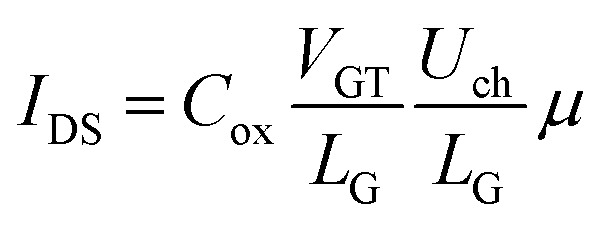
where *V*_GT_ is the overdrive voltage, *U*_ch_ is the voltage of channel under gate along source and drain direction, *L*_G_ is gate length. *R*_channel_ could be obtained through8
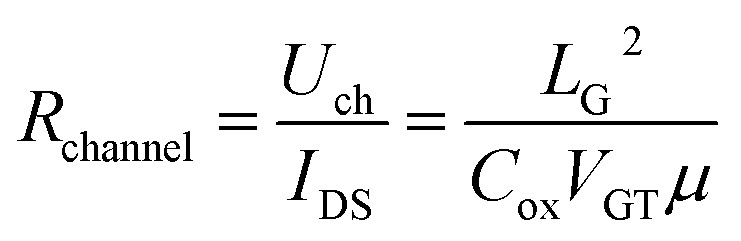


The final result could be brought out by bring Formula (6) back to Formula (1)9
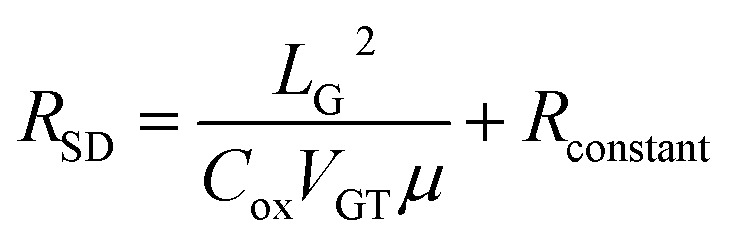


A linear relationship was established between *R*_SD_ and *μ*, where *R*_SD_ could be directly obtained from the measurement results. The mobility and non-gate resistance could be calculated from the slope and intercept, respectively.

**Fig. 3:**
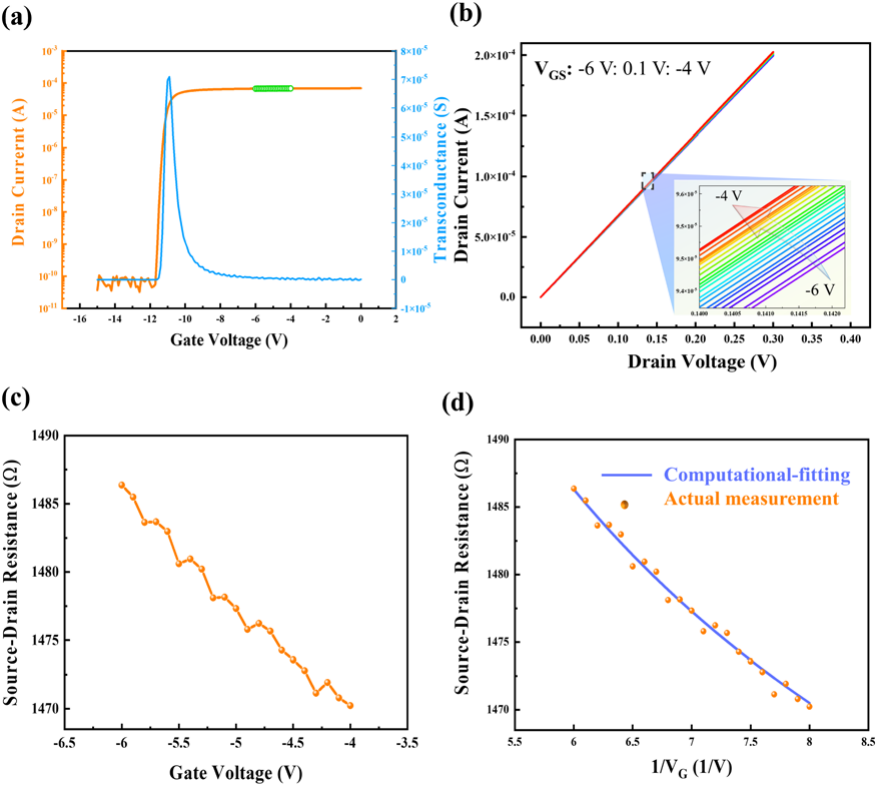
The fitting process of mobility and non-gate resistance. (a) Transfer characteristics measured at *V*_D_ = 0.1 V, (b) *I*_D_ measured at a small range of *V*_D_. (c) *R*_SD_ measured with gate voltage ranges from −6 V to −4 V. (d) A comparison of *R*_SD_ between fitting and measured results.

Fig. 3 illustrates in detail the computational-fitting method and the corresponding results. Initially, the transfer characteristics were measured at *V*_D_ = 0.1 V, and *V*_G_ ranging from −15 V to 0 V in 0.1 V increments, yielding a stable *I*_D_ and a corresponding gate voltage range. Then *R*_SD_ was measured based on the above-obtained *V*_G_ range, where *V*_GS_ changes from −6 V to −4 V with 0.1 V increments, and *V*_D_ ranges from 0 to 0.3 V to ensure the quasi-static conditions, allowing carrier mobility and electrical field in channel to be approximately constant.^26^ The final fitting results demonstrate that this HEMT device has a mobility of approximately 302.3 cm^2^ V^−1^ s^−1^, and a non-gate resistance of 1531 Ω.

Fig. 3 illustrates in detail the computational-fitting method and the corresponding results. Initially, the transfer characteristics were measured at *V*_D_ = 0.1 V, and *V*_G_ ranging from −15 V to 0 V in 0.1 V increments, yielding a stable *I*_D_ and a corresponding gate voltage range. Then *R*_SD_ was measured based on the above-obtained *V*_G_ range, where *V*_GS_ changes from −6 V to −4 V with 0.1 V increments, and *V*_D_ ranges from 0 to 0.3 V to ensure the quasi-static conditions, allowing carrier mobility and electrical field in channel to be approximately constant.^27^ The final fitting results demonstrate that this HEMT device has a mobility of approximately 302.3 cm^2^ V^−1^ s^−1^, and a non-gate resistance of 1531 Ω.

**Fig. 4:**
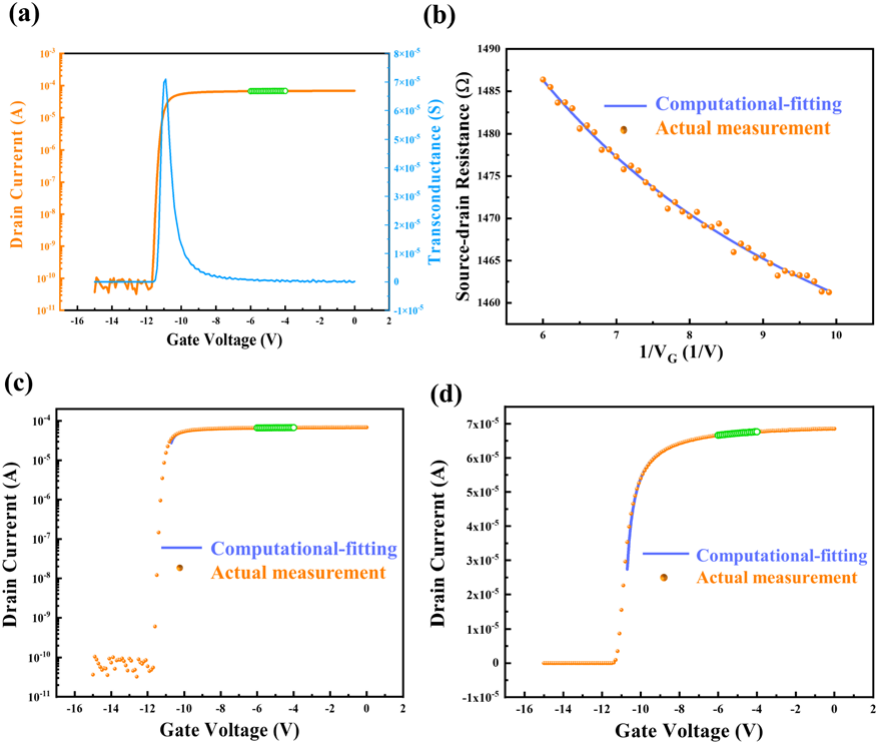
The feasibility of computational-fitting method. (a) and (b) show the computation and fitting process, in which mobility and non-gate resistance were obtained. (c) and (d) show the electrical characteristics in logarithmic and linear coordinates, respectively, which were reversely derived from the computational-fitting method to verify the accuracy.

A series of experiments were conducted to verify the feasibility and stability of the computational-fitting approach. The device mobility and non-gate resistance were calculated by fitting within the *V*_g_ range of −6 V to −4 V. The resulting values were then substituted into the fitted model (eqn (9)) to derive the electrical characteristics of the entire device. This allows for the derivation of the electrical characteristics of the device outside of this measurement range (*i.e.*, *V*_G_ outside of −6–−4 V) based on the calculated values. The transfer characteristic curve of the HEMT device back-propagated by this method is depicted in Fig. 4. The fitting characteristic curve shows high consistence with the actual measured result of the HEMT device, demonstrating that the computational-fitting method is reliable and also an effective method for accurately determining the electrical characteristics of HEMT devices beyond the measurement range.

**Fig. 5:**
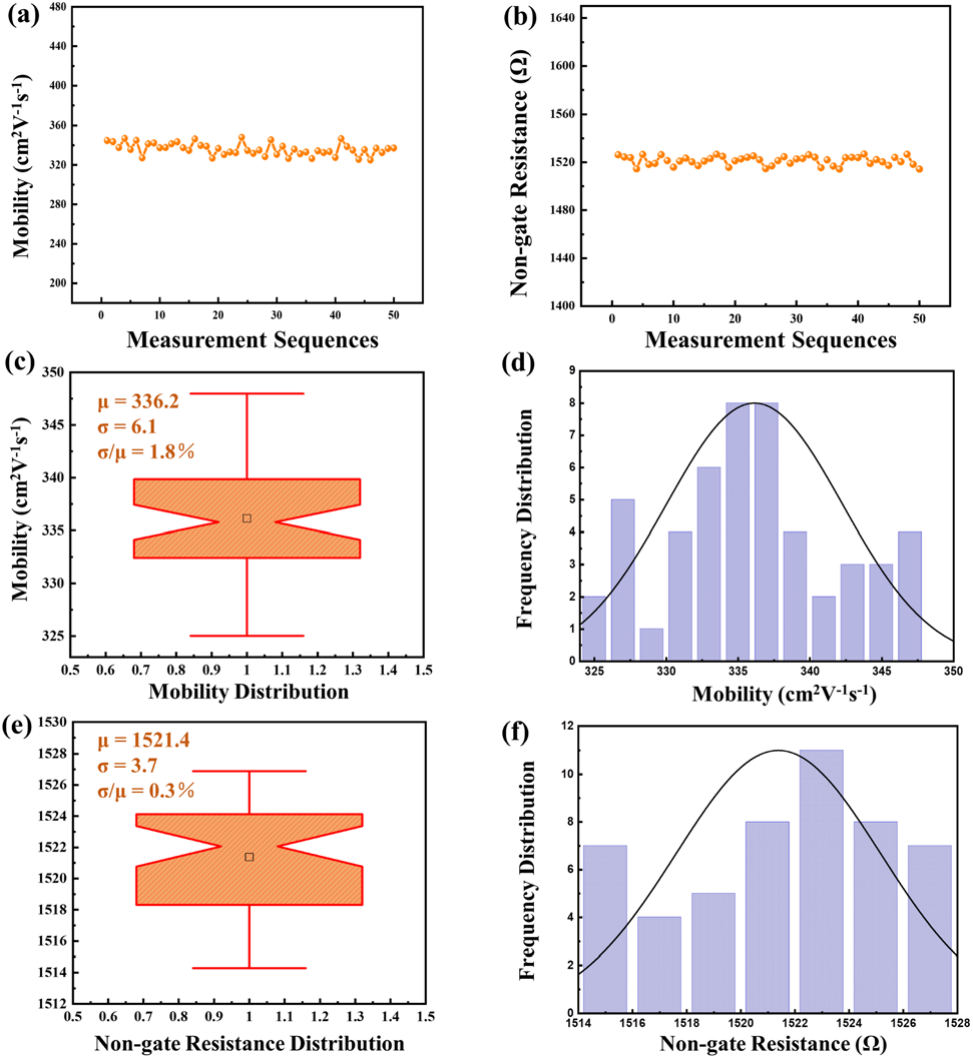
The reliability of computational-fitting method. (a) and (b) show the measurement results of mobility and non-gate resistance for 50 times. (c) and (e) show the distribution of mobility and non-gate resistance respectively using box plots. (d) and (f) show the normal distribution of mobility and non-gate resistance respectively.

Moreover, to ensure the robustness of the computational-fitting approach, 50 sets of carrier mobilities and non-gate resistances were extracted from the same HEMT device using the fitting method for 50 times, as shown in Fig. 5. The resulting 50 sets of mobility data exhibited a small standard deviation of 6.1 cm^2^ V^−1^ s^−1^ and a mean value is 336.2 cm^2^ V^−1^ s^−1^, with a concentrated data distribution and a fluctuation of only 1.8%. Similarly, the 50 sets of non-gate resistance data had a small standard deviation of 3.7 Ω and a mean value of 1521 Ω, with a fluctuation of only 0.3%. These results demonstrate that there is no correlation between the measurement series and the fitting results, validating the stability of the computational-fitting approach. Furthermore, 50 HEMT devices have been tested to evaluate robustness when accounting for device-to-device variation. The mean values for mobility and non-gate resistance are 301.6 cm^2^ V^−1^ s^−1^ and 1365.6 Ω, respectively. The standard deviation (sigma) for mobility and non-gate resistance stands at 7.1 cm^2^ V^−1^ s^−1^ and 4.9 Ω, exhibiting a mere 2.3% and 0.4% fluctuation, underlining the exceptional robustness of this method in accommodating device-to-device variations. Therefore, this method is reliable and can be applied to accurately determine the carrier mobilities and non-gate resistances of HEMT device.

## Conclusions

In summary, this work demonstrated an effective method for extracting carrier mobility from GaN HEMT device using a computational-fitting technique based on the fundamental current equation and actual electrical measurements. The proposed method achieves high accuracy, simplicity, and low cost compared to traditional methods. It is particularly useful in cases where the non-gate region is quite large and the effect of non-gate resistance cannot be ignored. The proposed computational-fitting method has also been verified to exhibit excellent reliability and stability without any negative impact on the calculation of GaN HEMT mobility. Therefore, it is a valuable method for accurately determining carrier mobility in such devices. Overall, this work presents a significant contribution to the field of GaN HEMT device characterization and provides a valuable tool for future research in this area.

## Author contributions

Kuan-Chang Chang: investigation, formal analysis, writing – original draft, writing – review & editing. Xibei Feng: data curation, validation, writing – review & editing. Huangbai Liu: data curation, visualization. Kai Liu: validation. Xinnan Lin: resources, project administration. Lei Li: conceptualization, methodology, writing – review & editing, funding acquisition.

## Conflicts of interest

There are no conflicts to declare.

## Supplementary Material
